# External aluminium supply regulates photosynthesis and carbon partitioning in the Al-accumulating tropical shrub *Melastoma malabathricum*

**DOI:** 10.1371/journal.pone.0297686

**Published:** 2024-03-20

**Authors:** Khairil Mahmud, Hedda Weitz, Ully H. Kritzler, David F. R. P. Burslem

**Affiliations:** 1 Faculty of Agriculture, Department of Crop Science, Universiti Putra Malaysia (UPM), Seri Kembangan, Selangor, Malaysia; 2 Institute of Bioscience, Biodiversity Unit, Universiti Putra Malaysia, Serdang, Selangor, Malaysia; 3 School of Biological Sciences, University of Aberdeen, Scotland, United Kingdom; 4 Department of Earth and Environmental Sciences, The University of Manchester, Manchester, United Kingdom; Bahauddin Zakariya University, PAKISTAN

## Abstract

Aluminium (Al) is toxic to most plants, but recent research has suggested that Al addition may stimulate growth and nutrient uptake in some species capable of accumulating high tissue Al concentrations. The physiological basis of this growth response is unknown, but it may be associated with processes linked to the regulation of carbon assimilation and partitioning by Al supply. To test alternative hypotheses for the physiological mechanism explaining this response, we examined the effects of increasing Al concentrations in the growth medium on tissue nutrient concentrations and carbon assimilation in two populations of the Al-accumulator *Melastoma malabathricum*. Compared to seedlings grown in a control nutrient solution containing no Al, mean rates of photosynthesis and respiration increased by 46% and 27%, respectively, total non-structural carbohydrate concentrations increased by 45%, and lignin concentration in roots decreased by 26% when seedlings were grown in a nutrient solution containing 2.0 mM Al. The concentrations of P, Ca and Mg in leaves and stems increased by 31%, 22%, and 26%, respectively, in response to an increase in nutrient solution Al concentration from 0 to 2.0 mM. Elemental concentrations in roots increased for P (114%), Mg (61%) and K (5%) in response to this increase in Al concentration in the nutrient solution. Plants derived from an inherently faster-growing population had a greater relative increase in final dry mass, net photosynthetic and respiration rates and total non-structural carbohydrate concentrations in response to higher external Al supply. We conclude that growth stimulation by Al supply is associated with increases in photosynthetic and respiration rates and enhanced production of non-structural carbohydrates that are differentially allocated to roots, as well as stimulation of nutrient uptake. These responses suggest that internal carbon assimilation is up-regulated to provide the necessary resources of non-structural carbohydrates for uptake, transport and storage of Al in *Melastoma malabathricum*. This physiological mechanism has only been recorded previously in one other plant species, *Camellia sinensis*, which last shared a common ancestor with *M*. *malabathricum* more than 120 million years ago.

## Introduction

Aluminium toxicity is an important limiting factor for crop productivity on acid soils globally [1–3]. Soil with an Al concentration of 2–5 ppm is considered toxic to many plants [3]. Therefore it is a paradox that positive effects of Al addition on the growth of a small number of wild plant species adapted to acid soils have also been reported [4–6]. The species that have been shown to respond positively to Al addition in terms of growth include both Al accumulators, such as *Camellia sinensis* [7–11] and *Melastoma malabathricum* [4,12–14] and non Al accumulators native to acid soils such as *Miscanthus sinensis* [15] and *Eucalyptus gummifera* [16]. These results suggest that although Al is a toxic element to most plants, it may act as a stimulant to the growth of others.

There are several existing hypotheses for the mechanism of Al-induced increases in plant growth. Research on the Al accumulating tropical shrub *Melastoma malabathricum* has shown that Al addition increases the production of root cortex cells and enhances root development [17], stimulates the uptake of N, P, K, Ca and Mg [14,18,19] and potentially alleviates Fe and H^+^ toxicity [20]. However more recent work on tea (*Camellia sinensis*) has shown that addition of 0.3 mM Al to plants growing in solution culture stimulates rates of stomatal conductance and photosynthesis, which suggests that the positive effect of Al on growth may be linked to enhanced rates of carbon assimilation [9,21].

To our knowledge the effects of Al addition on carbon assimilation and partitioning in Al accumulator plant species other than tea have not been examined. Seedlings of *M*. *malabathricum* grown in nutrient solutions showed increased relative growth rate, net assimilation rate and a shift in dry mass allocation from leaves to roots in response to an increase in Al concentration from 0 to 0.5 mM Al [4,12]. This combination of morphological and physiological changes provides indirect evidence that Al addition may also enhance the rates of photosynthesis in *M*. *malabathricum*, although this remains to be tested. In recent research we have shown that growth rates and tissue Al concentrations differ widely among different populations *M*. *malabathricum*, and that seedling growth rates are positively correlated with foliar nutrient and Al concentrations among populations [4]. If up-regulation of photosynthetic rates contributes to the positive growth response of *M*. *malabathricum* seedlings to Al then we expect a greater response among populations with inherently faster growth rates.

Al accumulation has a significant carbon cost resulting from the mechanisms of Al uptake, internal transport and detoxification. In *M*. *malabathricum*, uptake of Al is facilitated by secretion of polysaccharide-rich mucilage close to the root tips, which has a high affinity for trivalent cations such as Al^3+^ [19]. The Al taken up into the root apoplast is chelated with citrate during xylem transport, and then complexed with oxalate for storage in the apoplast and vacuole of leaves [20]. In tea (*Camellia sinensis*), Al chelates with oxalate in the root apoplast, but the ligand changes to citrate during xylem loading and then to an Al-catechin complex for storage in the vacuoles of leaf cells [9,22,23]. These mechanisms of Al uptake, internal transport and detoxification by Al accumulators require synthesis of carbohydrates or other carbon-rich compounds that are ultimately derived from photosynthesis. Therefore it is potentially significant that stomatal conductance and photosynthesis of tea seedlings responded positively to the addition of Al, as these processes have the capacity to enhance the pool of available carbon metabolites [9]. It follows that stimulation of photosynthesis in response to Al addition may represent a fundamental component of the mechanism underlying evolution of the Al accumulation trait. If this hypothesis is correct then Al addition is predicted to directly stimulate photosynthesis and concentrations of soluble non-structural carbohydrates in other Al accumulators, but is less likely to trigger increased concentrations of structural carbon compounds such as cellulose, hemicellulose and lignin except where these are directly related to processes involved in Al uptake, transport or storage. For example, Al addition has been shown to decrease lignin concentrations in the roots of tea plants, but not in stems or leaves [9,24].

The aim of this study was to investigate the effects of Al addition on nutrient uptake, photosynthesis and respiration rates, and partitioning of carbon between structural and non-structural components for seedlings of two populations the tropical shrub *M*. *malabathricum* L. (Melastomataceae) that have shown contrasting growth rates and responses to Al addition in previous research [4]. This species was chosen because it is a known Al accumulator plant and previous studies have suggested that its growth may respond positively to Al addition under certain growing conditions [4,12,17,25]. *M*. *malabathricum* is a widespread small bird-dispersed shrub commonly found in a range of natural vegetation types as well as disturbed land, secondary forest and roadsides, with a range extending across South and South-East Asia, China, Taiwan, Australia, and the South Pacific [17,26]. In some localities, including Malaysia, the leaves and roots of *M*. *malabathricum* are used for medicinal purposes [27]. This paper extends our earlier work by addressing some of the underlying mechanisms that determined the differential growth responses to Al among populations from the perspective of carbon assimilation and partitioning. The specific hypotheses addressed by this study were as follows.

- Increases in growth rate in response to Al addition are associated with coordinated up-regulation of photosynthesis and respiration rates, and increased tissue concentrations of non-structural carbohydrates.
- Changes in concentrations of non-structural carbohydrates and structural carbon fractions in response to Al addition are expressed more strongly in roots than above-ground tissues.
- Individuals from a population of fast-growing *M*. *malabathricum* have a greater capacity to up-regulate rates of photosynthesis and respiration in response to exogenous Al supply than individuals from a slow-growing population. These differences will also be manifested by variation among populations in tissue concentrations of nutrients and non-structural carbohydrates.

## Materials and methods

### Seed collection

Fruits of *Melastoma malabathricum* L. were collected from a total of 18 populations across Peninsular Malaysia. Experimental research on the progeny of these populations growing under uniform growing conditions showed that they displayed consistent variation in growth rates that were associated with nutritional characteristics including tissue Al concentrations [4,18]. For the research described in this paper we selected populations in the states of Kedah and Perlis, Peninsular Malaysia, which displayed the slowest and fastest relative growth rates, respectively, among the 18 Malaysian populations examined previously [4]. At each site, a total of 10–12 fruits was collected from at least three individuals (range 3 to 5 individuals) and mixed together to create a bulk sample for each population. The seeds were extracted from the partly opened fleshy fruits in distilled water, rinsed with distilled water several times, then filtered and left to air-dry in an air-conditioned laboratory at the Universiti Sultan Zainal Abidin, Malaysia. Air-dried seeds were transported to the University of Aberdeen, U.K., for experimental work. *M*. *malabathricum* is not listed on CITES or by the IUCN as a threatened species, therefore export of seeds to the UK did not require a permit.

### Growing conditions and analyses

A total of 216 seeds was sown in batches of six on the surface of Daishin agar (0.5 g agar 100 ml−1 with 50% Hoagland’s nutrient solution) in sterilized 0.5 μl Eppendorf tubes. These tubes were distributed individually among 36 sterilized containers containing 50% Hoagland solution (for composition see [4]). The bottom 2 mm of the Eppendorf tubes had been removed to enable growth of the *M*. *malabathricum* roots into a nutrient solution. The containers were placed in a growth chamber and were re-randomized weekly. The growth chamber was set to deliver a temperature of 27°C and 12/12 h light/dark photoperiod with an irradiance of 200–250 μmol m^-2^ s^-1^. The pH of the nutrient solutions was checked daily and adjusted to 4.0 using 1.0 M NaOH or 1.0 M HCl, and the nutrient solutions were renewed weekly throughout the growing period following [4]. The seeds germinated after 7–9 days, and 14 days after sowing the seedlings were thinned to one per Eppendorf tube by randomly selecting excess surviving individuals for removal.

Twenty eight days after germination (five weeks from sowing), the nutrient solutions in the containers were amended by the addition of 0 mM, 0.5 mM or 2.0 mM AlCl_3_ to achieve six replicates of each Al treatment per population. The chemical equilibrium programme MINTEQ ver 3.1 [28,29] was used to estimate speciation of Al ions and compounds in these solutions under the assumptions of this model. The results suggested that 85.4% and 68.0% of the Al in the 50% Hoaglands solutions amended with 0.5 mM and 2.0 mM AlCl_3_, respectively, would have been complexed or precipitated with either sulphate or phosphate, yielding estimated equilibrium concentrations of free Al^3+^ in the solutions of 68.8 μM (0.5 mM treatment) and 605.2 μM (2.0 mM treatment). The reliability of these estimates is uncertain because uptake of ions and exudation of both organic and inorganic constituents by the seedlings growing in the solutions would have had effects that are not accounted for by chemical speciation models. Despite this uncertainty, the ~9-fold difference in estimated equilibrium concentrations of Al^3+^ suggests that the difference in starting concentrations of Al^3+^ between these treatments would have translated into a real difference in exposure of the plants to free Al, and this inference is supported by measured differences in tissue Al concentrations in response to treatments (see below).

Measurements of photosynthesis and respiration rates were made ten weeks after the start of Al treatments. The plants were harvested 14 weeks after sowing following the completion of photosynthesis and respiration measurements. During the harvest, a small fraction (approximately 4 cm^2^) of the fresh leaf used for gas exchange measurements, as well as small sections of fresh stem and root material, were transferred to sterile containers and stored immediately at -70° C prior to analysis of tissue element and non-structural carbohydrate concentrations as described below. The remaining material was divided into stems, leaves and roots, and dried and weighed as above to determine final total dry mass (growth results reported in [4]).

Measurements of light saturated photosynthetic rate (A_sat_) and dark respiration rate (R_d_) were made on the youngest fully expanded leaf on each plant selected in random sequence over three days just prior to the final harvest using a closed leaf chamber attached to a LI-6400XT portable photosynthesis system (LICOR, Lincoln, NE, USA). The A_sat_ measurements were conducted during the period 0900–1130 h, and the R_d_ measurements were conducted during the period 1600–1800 h after the plants had been wrapped in black plastic for one hour. Photosynthesis was recorded under constant high light (PPFD 2000 μmol m^-2^ s^-1^), ambient CO_2_ concentration (400 ppm), a block temperature of 30°C and a leaf temperature of 25°C. Measurements were recorded only when steady state gas exchange had been maintained for at least two minutes.

To determine elemental concentrations in plant leaves, a fragment of 0.5–1.0 cm^2^ of the young and fully expanded leaf material was removed from the margin of three randomly selected individuals per population x Al treatment combination during the harvest. This material was cut in transverse section, washed in deionized water and placed in a 50μl Teflon tube. Sections of fresh stem and root material of approximately 15 mm^2^ size were transferred to sterile containers and stored immediately at -70° C prior to analysis of tissue element and non-structural carbohydrate concentrations. Samples for elemental analysis were dried in an oven at 88°C for 20–22 hours, then digested using 70% nitric acid (HNO_3_) and analyzed by inductively-coupled plasma mass spectrometry (NexION 300D, ICP Mass Spectrometer, PerkinElmer, USA). This analysis yielded tissue concentrations of Al, P, K, Ca and Mg.

Concentrations of soluble sugars and starch were made on fresh stem and root samples taken from each individual and from the leaves used for gas exchange measurements (n = 36 per plant part). These samples were collected at the time of the final harvest and immediately frozen at -70°C to halt enzymatic activities. The samples were freeze-dried overnight and ground to a particle size of <0.15 mm [30]. Soluble sugars were measured in the supernatant of extractions of 5 mg sub-samples using 80% ethanol, while the pellet remaining after extracting the soluble component was digested for 16 h with 0.5% amylo-glucosidase solution in acetate buffer at 50°C. Quantification of soluble sugars in both fractions was conducted using the phenol-sulphuric acid reagent in a 96-well microplate reader (Biochrom EZread 400, USA) measuring absorbance at 490 nm [31] with eight blanks per plate. The calibration curve was constructed using 0, 300, 600, 900, 1200 and 1500 ppm glucose solution standards. Total non-structural carbohydrate (TNC) was determined as the sum of soluble sugars and starch [30,32–34].

Concentrations of cellulose, hemicellulose and lignin were determined in 500 ± 5 mg sub-samples of oven-dried and ground leaf, stem and root material using an ANKOM 2000 fibre analyser (ANKOM Technology, USA). Samples were placed separately in filter bags and rinsed in successive steps with neutral detergent solution, acid detergent solution and 72% sulfuric acid. These reagents extract cellulose, hemicellulose and lignin components of plant cells, respectively, and intermediate drying and weighing steps allow these quantities to be estimated.

### Statistical analysis

All analyses were conducted using R version 3.3.1 [35]. Two way analysis of variance (ANOVA) was conducted in order to investigate the significance of differences in physiological measurements, foliar nutrient concentrations, and fibre and non-structural carbohydrate contents between populations, treatments and the interaction between these variables. To characterise the extent of coordination among traits for plants growing in the absence or presence of Al, principal components analyses (PCAs) were conducted separately for plants grown in the treatments with 0 mM Al (12 plants) and either 0.5 mM or 2.0 mM Al (24 plants) in the growing medium. The traits included in these PCAs were light saturated photosynthetic rate (A_sat_), dark respiration rate (R_d_), whole-plant concentrations of P, K, Ca and Mg, root concentrations of soluble sugars, starch, cellulose, hemicellulose and lignin, and root: shoot ratio, measured in all cases at the final harvest. All variables were centred and standardised prior to running the PCAs. To determine how coordinated variation among these traits affected plant growth rates, data on final dry mass were fitted to linear regression models with independent variables determined by scores along the first and second principal component axes for each set of plants (i.e. those grown in the absence or presence of Al). Anderson Darling tests were used to confirm that residuals were normally distributed after model fitting in all cases.

## Results

### Photosynthesis and respiration

**Fig 1:**
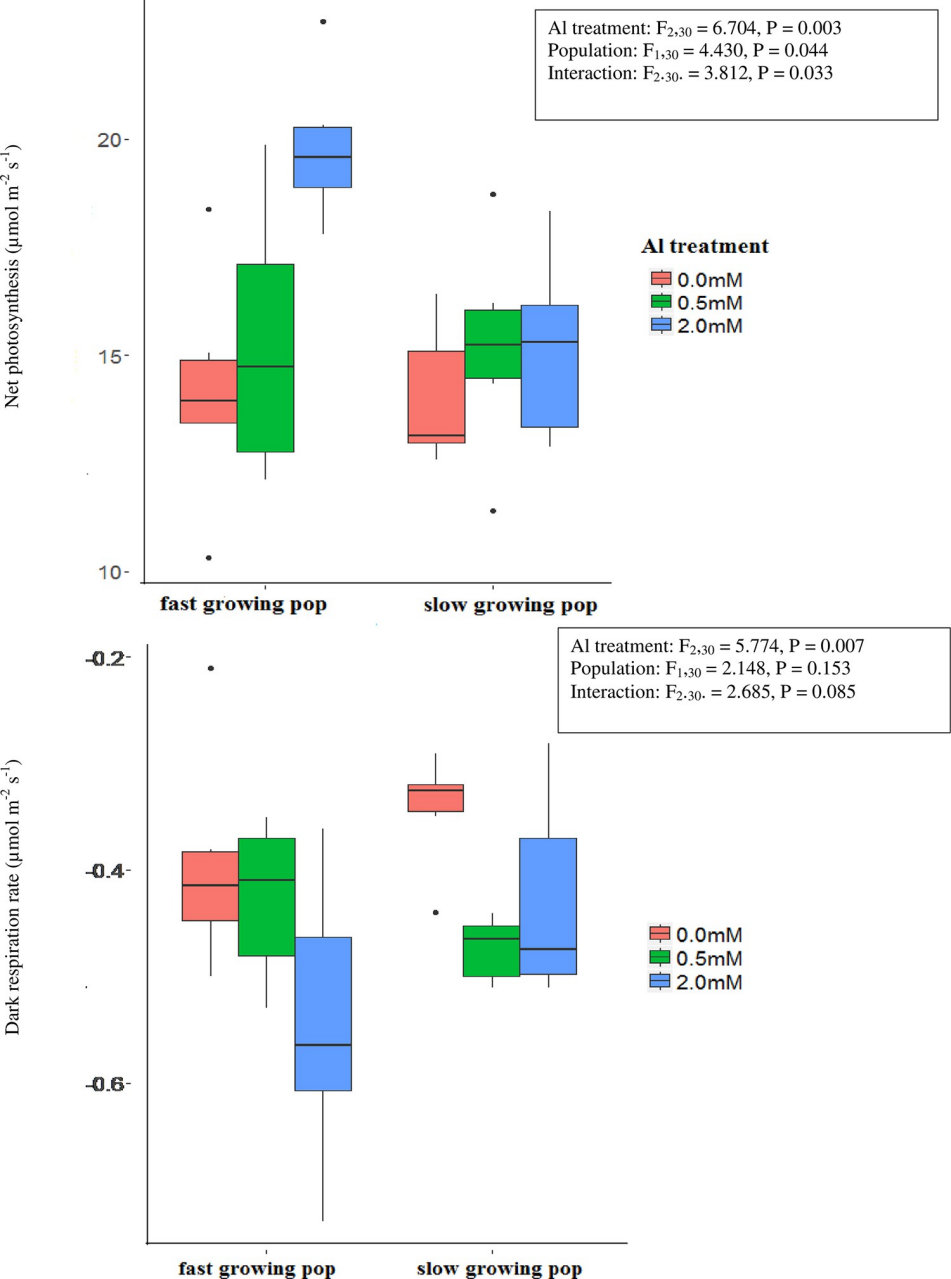
Boxplots of (a) net photosynthesis (μmol m^-2^ s^-1^) at 400 μm mol CO_2_ (A_*sat*_) and (b) dark respiration rate (μmol m^-2^ s^-1^) of slow and fast growing populations of *M*. *malabthricum* seedlings grown for 10 weeks in nutrient solutions containing 0 mM, 0.5 mM, or 2.0 mM AlCl_3_. The boxed keys present F and p values from two-way analyses of variance that test the significance of differences among Al treatments, populations and the interaction between Al treatments and populations.

Seedlings grown with Al in the nutrient solution displayed higher rates of photosynthesis (Fig 1A) and dark respiration (Fig 1B) after 10 weeks than seedlings grown in the absence of Al. The differential response of photosynthesis to Al treatments between the two populations was highly significant (Fig 1A): the mean increase in response to the presence of Al was 7.5% (0.5 mM Al solution) and 46.7% (2.0 mM Al solution) relative to the control for seedlings of the fast-growing population, while the equivalent increases for the slow-growing population were 24.1% and 18.2% respectively. The increase in dark respiration rate in response to the presence of Al was expressed equally in both populations, and there was only limited evidence of a differential response to Al treatments between the two populations (Fig 1B).

### Element concentrations in response to Al addition

**Fig 2:**
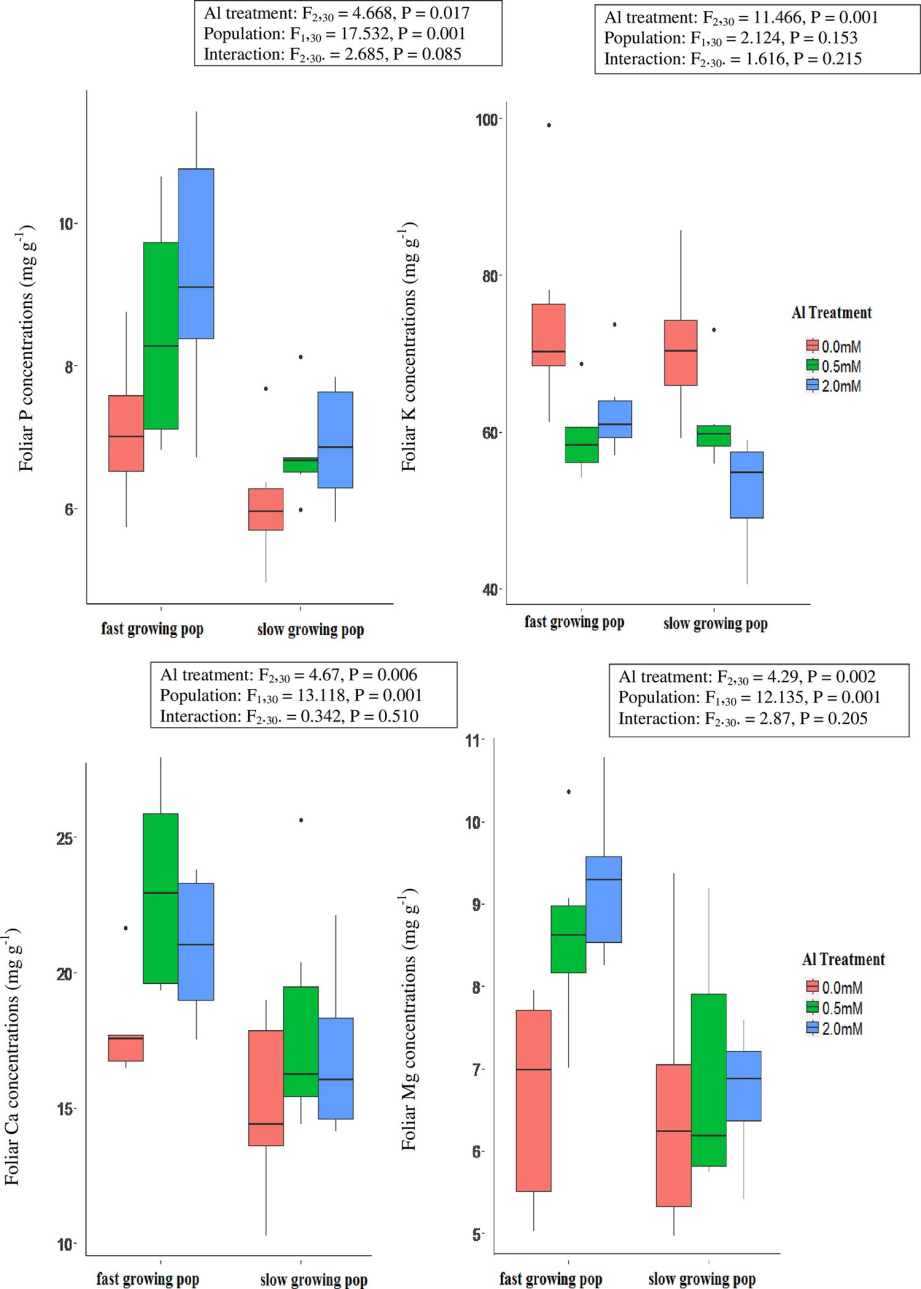
Boxplots of foliar P, K, Ca and Mg concentrations (mg g^-1^) of fast and slow growing populations of *M*. *malabathricum* seedlings grown for 10 weeks in nutrient solutions containing 0 mM, 0.5 mM, or 2.0 mM AlCl_3_. The boxed keys present F and p values from two-way analyses of variance that test the significance of differences among Al treatments, populations and the interaction between Al treatments and populations.

Al concentrations were very low (≤ 0.40 mg g^-1^) in tissues of plants that had been grown without addition of Al to the nutrient solution and were higher for plants that had been grown in nutrient solutions containing 2.0 mM than 0.5 mM Al for all tissues (S1 Table in S1 Appendix). The differential Al concentration in response to growth in the solution with the higher Al concentration was greatest for roots (+82% for the slow-growing population and +119% for the fast-growing population). Growth of seedlings in nutrient solutions containing Al tended to increase concentrations of P, K, Ca and Mg in plant tissues over values in the no-Al control, although responses varied among organs and populations (Fig 2 for leaves, S1 and S2 Figs in S1 Appendix for stems and roots respectively, and S1 Table in S1 Appendix for all data). In both leaves and stems, concentrations of P, Ca and Mg increased in response to the addition of Al to the nutrient solution, while K concentrations increased in stems but declined in leaves in response to Al addition (Fig 2 and S1 and S2 Tables in S1 Appendix). Concentrations of P were consistently higher for the fast growing population than the slow growing population in both leaves and stems, while concentrations of K were higher for the fast growing population in stems but did not differ between the two populations in leaves. Concentrations of both Ca and Mg in leaves were also higher for the fast growing population than the slow growing population (Fig 2), but in stems Ca concentrations did not differ between the populations and concentrations of Mg were lower for the fast growing population (S2 Table in S1 Appendix, S2 Fig in S1 Appendix). Inclusion of Al in the nutrient solution increased the concentrations of P, K and Mg in root tissue, and values of root K concentration were lower for the fast growing population than the slow growing population (S3 Fig in S1 Appendix). There was no evidence that the magnitude of changes in elemental concentrations in response to Al addition differed between the two populations (S2-S4 Tables in S1 Appendix).

### Cellulose, hemicellulose and lignin concentrations

**Fig 3:**
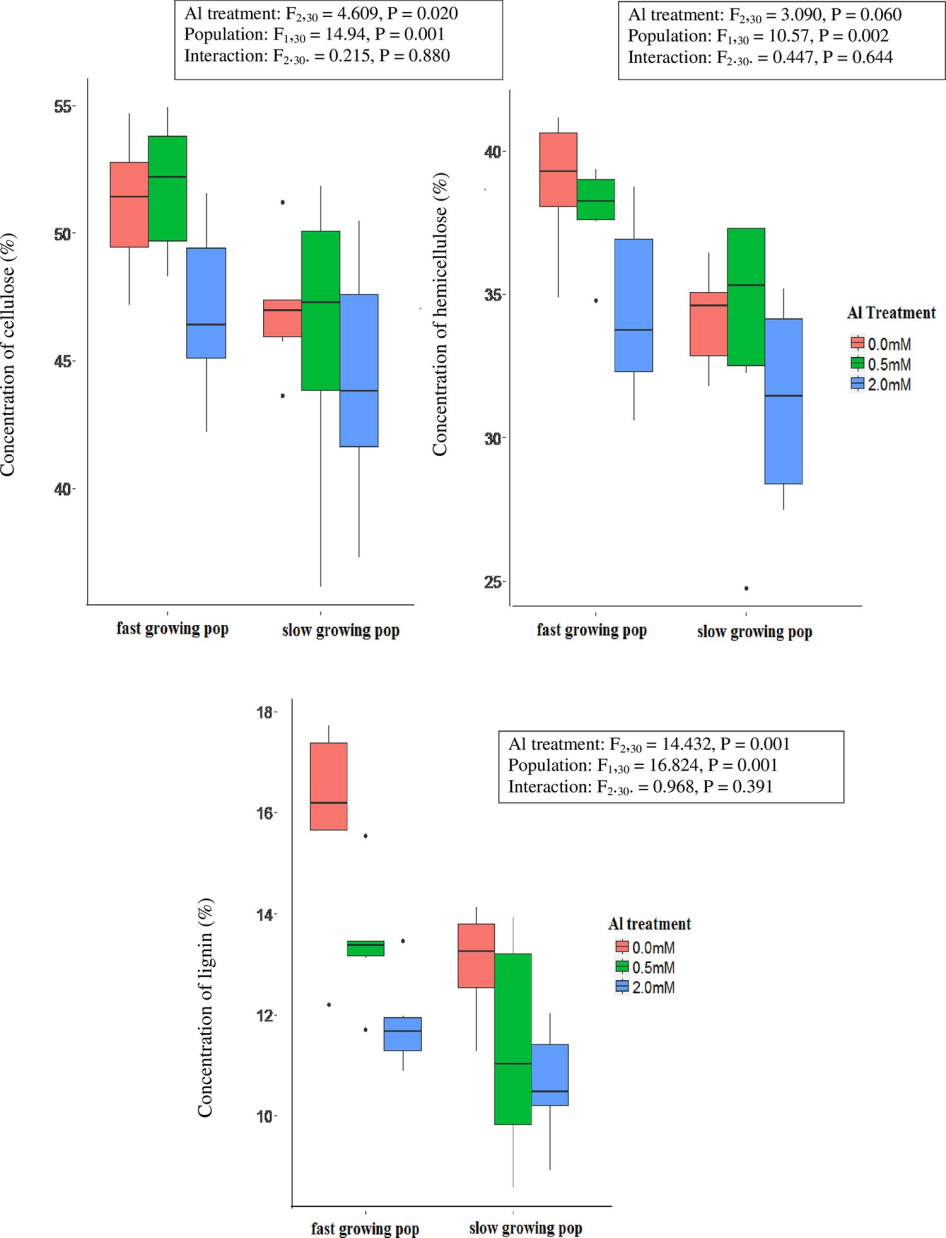
Boxplot of percentage concentrations of cellulose, hemicellulose and lignin in the roots of slow and fast growing populations of *M*. *malabathricum* seedlings grown for 10 weeks in nutrient solutions containing 0 mM, 0.5 mM, or 2.0 mM AlCl_3_. The boxed keys present F and p values from two-way analyses of variance that test the significance of differences among Al treatments, populations and the interaction between Al treatments and populations.

There were no differences among Al treatments or between populations in the concentrations of cellulose or lignin in either leaves or stems (S4-S6 Tables in S1 Appendix), but both these fractions declined significantly in root tissues in response to Al addition (Fig 3) and there was a parallel decline in hemicellulose concentrations in roots that was marginally non-significant (F_2,30_ = 3.09, P = 0.060, S4 Table in S1 Appendix). The concentrations of all three of these fractions in roots were higher for the fast growing than the slow growing population in all Al treatments, but there was no evidence that the percentage reduction in response to Al addition differed between the two populations (Fig 3). Hemicellulose concentrations in leaves increased by 2.8–4.2% in response to the inclusion of Al in the nutrient solution, but this response was similar between populations and was not expressed in stem tissue (S3 and S4 Figs in S1 Appendix).

### Non-structural carbohydrates

**Fig 4:**
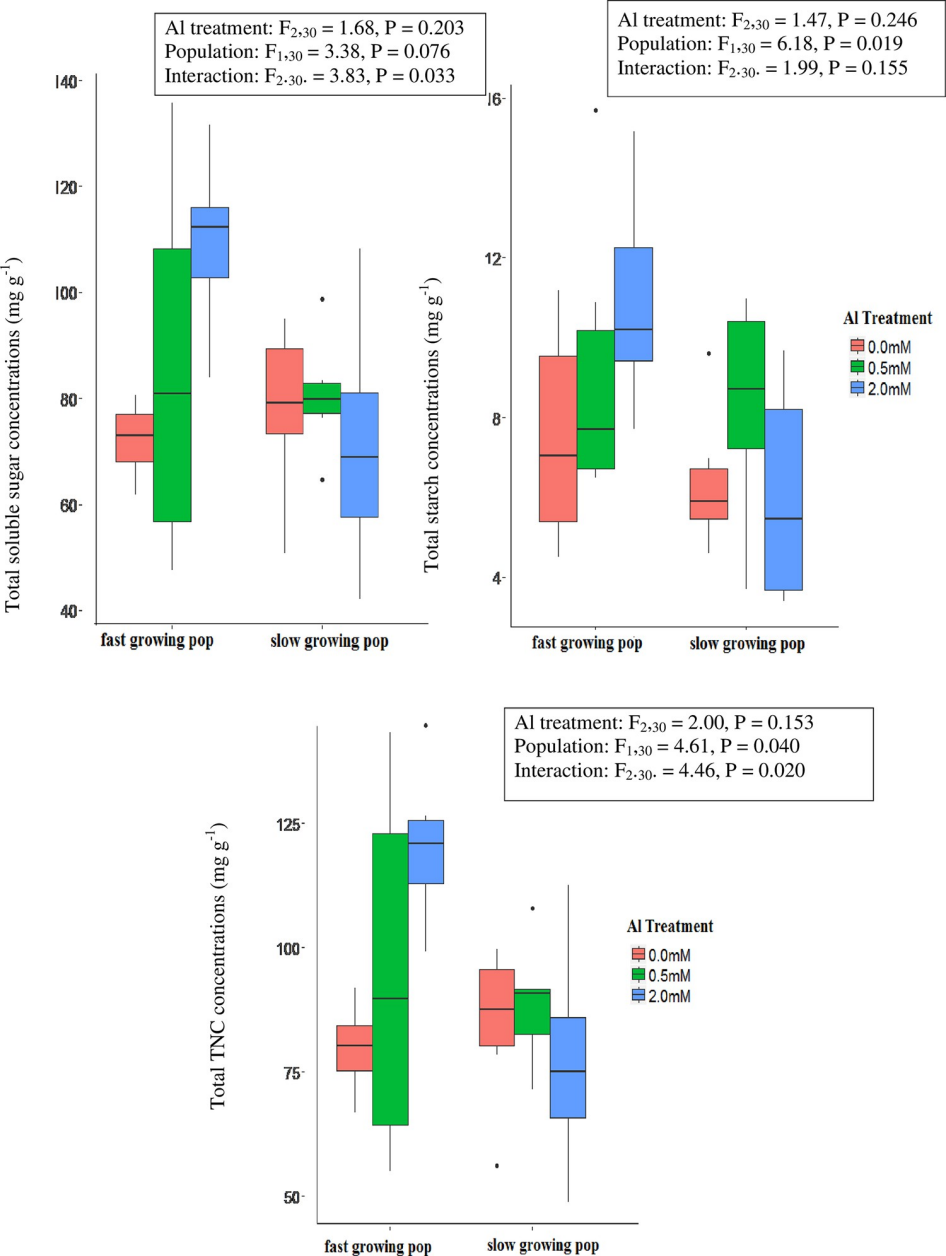
Boxplots of soluble sugar, starch and total non-structural carbohydrate (TNC) concentrations (mg g^-1^) in whole plants for seedlings of slow and fast-growing populations of *M*. *malabathricum* grown for 10 weeks in nutrient solutions containing 0 mM, 0.5 mM, or 2.0 mM AlCl_3_. The boxed keys present F and p values from two-way analyses of variance that test the significance of differences among Al treatments, populations and the interaction between Al treatments and populations.

Across all Al treatments total non-structural carbohydrate (TNC) concentrations for whole plants were higher in seedlings of the fast-growing population than the slow-growing population, which reflected a greater concentration of starch and a marginally non-significantly (F = 3.38, P = 0.076) greater concentration of soluble sugars in the fast-growing population (Fig 4). However, this difference between populations was driven by an increase in soluble sugar concentration when 2.0 mM Al was added to the nutrient solution for seedlings of the fast-growing population but not those of the slow-growing population (Fig 4). For the fast-growing population, addition of 0.5 mM Al to the nutrient solution had a much lower magnitude of effect on soluble sugar concentrations than the 2.0 mM Al addition treatment, and Al treatments did not affect total starch concentrations for either population. When these whole-plant effects were disaggregated to compare variation among plant tissues, it is apparent that the responses to Al and between the populations are largely driven by differential allocation of soluble sugars to roots, which were 33.5% greater, overall, for seedlings of the fast-growing population than the slow-growing population, and increased by 44.5% and 45.3% for seedlings grown in the presence of 0.5 and 2.0 mM Al, respectively, relative to the control (S5 Fig in S1 Appendix, S7 Table in S1 Appendix). The concentration of starch was greater for seedlings of the fast-growing population than the slow growing population in roots and starch concentrations also increased in response to the presence of Al in the nutrient solution, but only in stem tissue (S6 Fig in S1 Appendix, S8 Table in S1 Appendix).

### Effects of Al on trait coordination and growth

**Fig 5:**
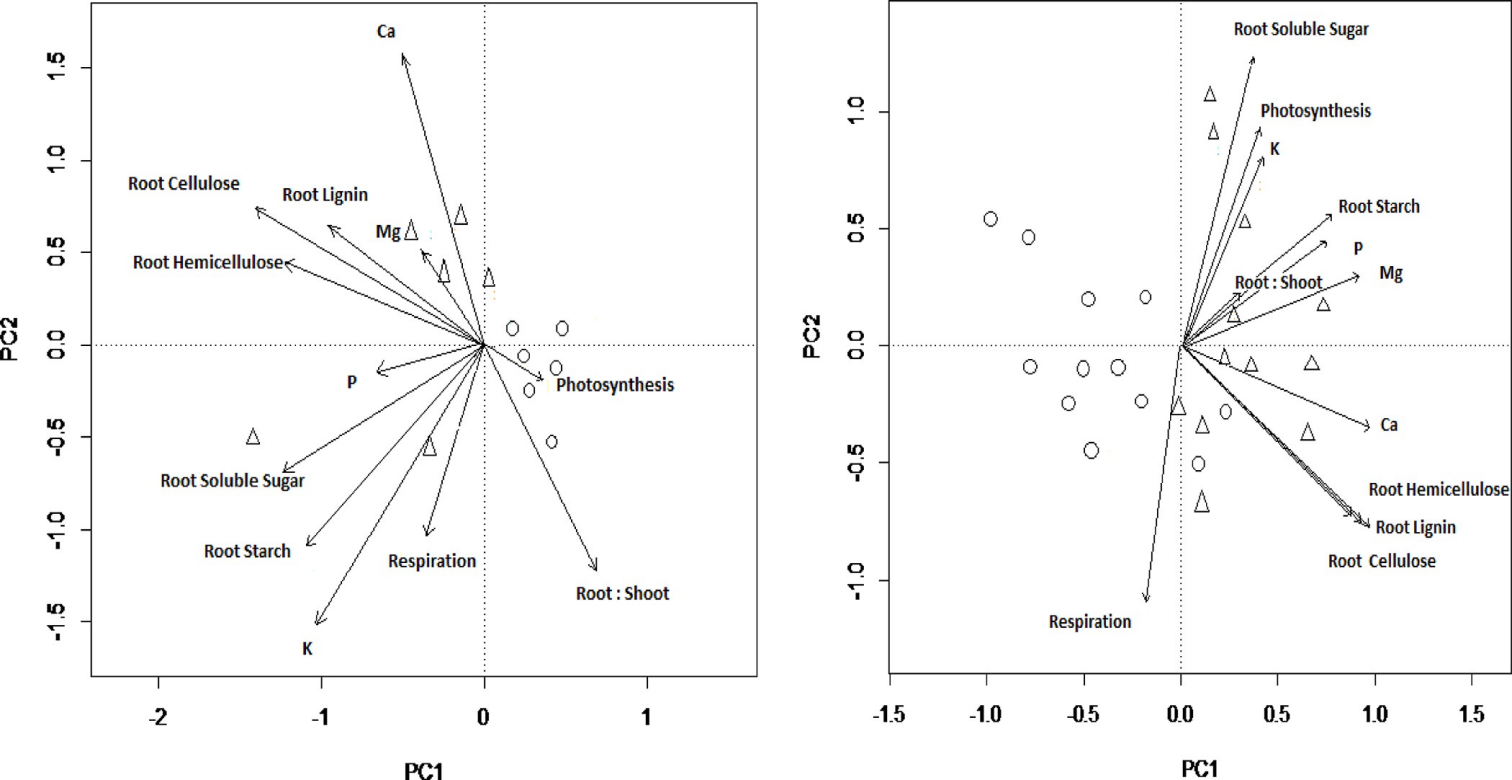
**a.** Biplot showing the distribution of six individuals from each of two *M*. *malabathricum* populations (circles, slow growing populations; triangles, fast growing population) along principal component axes 1 and 2 from a PCA summarising variation in foliar nutrient concentrations and physiological variables for seedlings grown for 28 days without Al addition (0 mM AlCl_3_). PC1 and PC2 accounted for 36% and 54% of the total cumulative proportion of variation respectively. The arrows show the loadings of each variable on the first two principal component axes. **b**. Biplot showing the distribution of 12 individuals from each of two *M*. *malabathricum* populations (circles, slow growing populations; triangles, fast growing population) along principal component axes 1 and 2 from a PCA summarising variation in foliar nutrient concentrations and physiological variables for seedlings grown for 28 days with Al addition (0.5 and 2.0 mM AlCl_3_). PC1 and PC2 accounted for 33% and 56% of the total cumulative proportion of variation respectively. The arrows show the loadings of each variable on the first two principal component axes.

**Fig 6:**
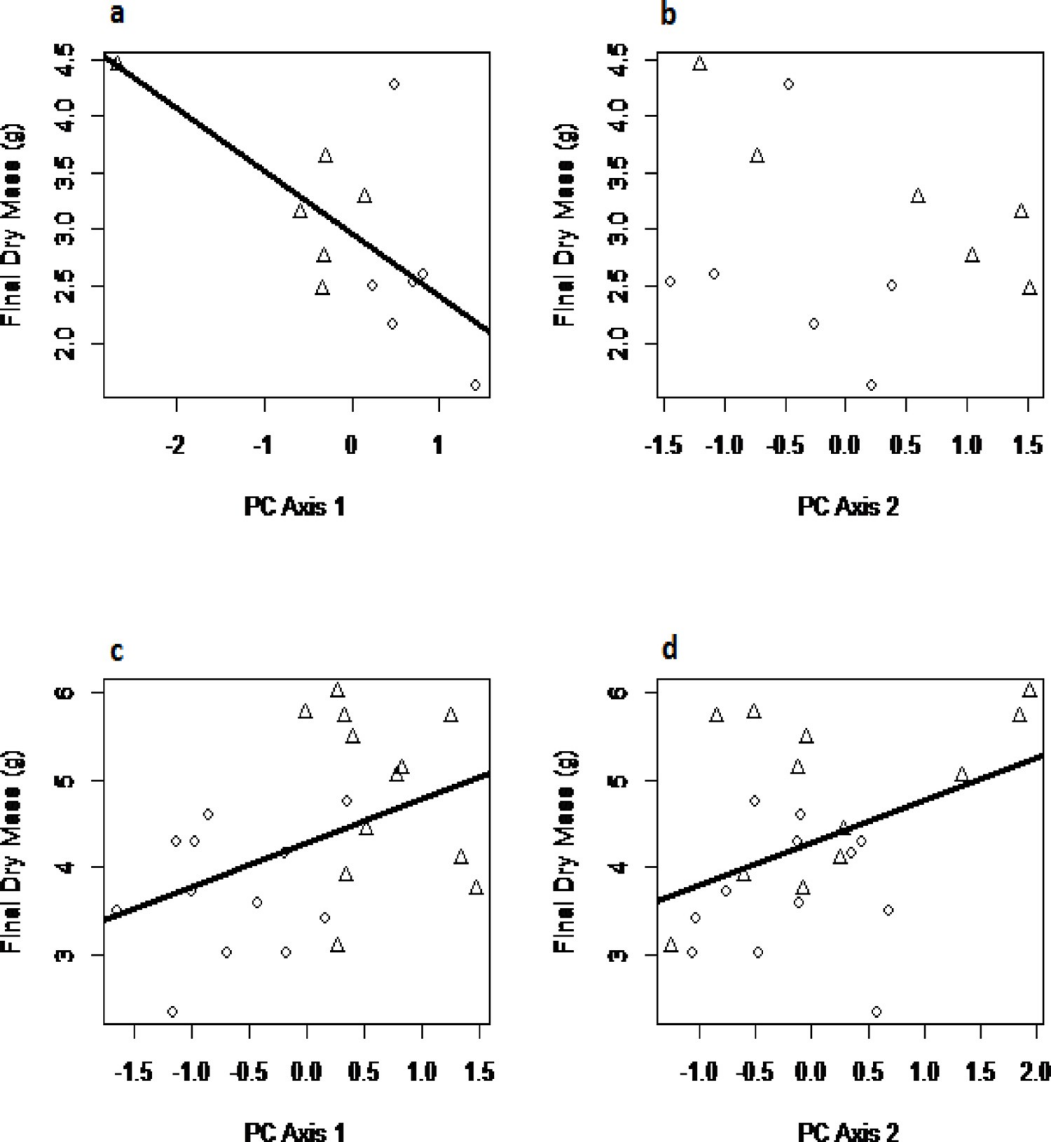
The relationships between the first (a, c) and second (b, d) axes from the PCAs displayed in Fig 5 summarising variation in physiological traits on plant total dry mass at the final harvest: (a, b) without Al addition to the nutrient solution, (c, d) with Al supplied as 0.5 or 2.0 mM AlCl_3_. Fitted lines (a, b and c) are from multiple linear regression models using coefficients presented in S11a and S11b Table in S1 Appendix.

A principal components analysis (PCA) summarising variation in 12 physiological and carbon partitioning traits among plants grown without Al addition (Fig 5A) displayed a first axis explaining 36.1% of variation that reflected correlations among the concentrations of non-structural carbohydrates, fibres and lignin in roots (loadings -0.61 to -0.88), as well as the K concentration of whole plants (loading -0.64, S9 Table in S1 Appendix). The second axis, explaining 17.5% of variation, reflected an inverse correlation between whole-plant K and Ca concentrations (Fig 5A, S9 Table in S1 Appendix). In a multiple linear regression, plants with low values along the first component (i.e. possessing higher non-structural carbohydrate, lignin and fibre concentrations in roots and higher whole plant K concentration) had higher dry mass at the end of the experiment (F = 8.56, P = 0.017), but scores along the second axis did not explain final dry mass (Fig 6A and 6b; S10 Table in S1 Appendix).

An equivalent PCA summarising variation among 12 traits for the plants grown in the two treatments containing Al in the growing medium (Fig 5B) displayed a first axis explaining 32.9% of variation (S11 Table in S1 Appendix). This axis reflected positive correlations among root fibre, lignin and starch concentrations (loadings 0.66–0.91), and whole-plant concentrations of P (0.71), Ca (0.90) and Mg (0.85). The second PC axis, which explained 23.2% of variation, captured variation in whole-plant K concentration (loading 0.61), light-saturated photosynthesis (0.69), respiration rate (-0.86) and soluble sugar concentration in roots (0.91). A multiple regression model demonstrated significant positive relationships between scores along both principal components and final dry mass (Fig 6C and 6D; S12 Table in S1 Appendix).

## Discussion

### Physiological responses to Al addition

Net photosynthetic and dark respiration rates of *M*. *malabathricum* increased after ten weeks of growth in treatments containing Al relative to the no-Al control treatment. These results provide a mechanistic explanation for the increase in growth rate of these seedlings over ten weeks in response to Al addition to the nutrient solutions [4]. This study builds on significant experimental support for the idea that some plants can display a positive growth response to low concentrations of Al in growth media [8,11,13,36]. The only other plant demonstrated to show an increase in photosynthetic rate in response to Al addition is tea (*Camellia sinensis*), which responded positively to the presence of 0.3 mM Al in terms of photosynthetic rate and also increased chlorophyll and carotenoid concentrations in young leaves [9,37]. An increase in photosynthetic and growth rates was associated with an increase in stomatal conductance in tea plants when Al was added at concentrations in the range 15–200 μM [21]. For tea, increases in concentrations of chlorophyll a and chlorophyll b occurred in young leaves, which also contain higher Al concentrations. It is possible that Al-induced increases in the uptake of nutrients such as Ca and Mg, which are required for mitochondrial metabolism and as a constituent of chlorophyll in plants, may contribute to the increased photosynthetic rates of *M*. *malabathricum* and tea plants in response to Al addition, although this hypothesis requires further research [35,38].

The addition of Al increased dark respiration rate of *M*. *malabathricum* seedlings in this study. The higher respiration rate may be linked to Al-induced organic acid production, which can enhance respiratory metabolism in plant cells [39,40]. The up-regulation of organic acid production in response to exposure to Al has been implicated as a mechanism of Al tolerance and accumulation in Al accumulator plants [1,39]. For example, organic acids bind to and detoxify Al^3+^ ions [7,23] and contribute to enhanced nutrient uptake by roots [8,34]. These mechanisms of Al tolerance and accumulation require energy, and up-regulation of respiration rate may serve the function of increasing the supply the ATP to power these metabolic processes [38,40].

Seedlings grown in the presence of Al had consistently higher concentrations of P and Mg in all plant tissues, K in stems and roots, and Ca in leaves and stems, when compared to plants in the control (0 mM Al) treatment. These results support previous research on hydroponically grown Al accumulators, demonstrating positive effects of Al on uptake of P in *M*. *malabathricum* [13,14,17,18] and Ca in both *M*. *malabathricum* [12,14,17,18] and tea [8,11]. Al has also been shown to increase the uptake of P, K, Mg, Ca and N in *Camellia oleifera* [8] and *Fagopyrum esculentum* [41]. It has been suggested that an increase in P uptake in response to an increase in Al concentration in the nutrient solution (from 0.032 mM to 0.2 mM) contributed to an enhanced relative growth rate of *M*. *malabathricum* in the higher Al concentration treatment [20]. Our results partially support this finding, as P uptake increased in response to raising the Al concentration in the nutrient solution from 0.5 mM to 2.0 mM. This increase in tissue P concentrations may have contributed directly to the increase in growth of *M*. *malabathricum* seedlings, or the association of P concentration and growth may be an indirect outcome of changes in root system size and morphology that were triggered by Al addition. Further research is required to uncouple these pathways of causation linking Al addition to nutrient uptake and stimulation of growth rate.

### Carbon partitioning

The addition of Al reduced lignin concentration in the roots of *Melastoma malabathricum* seedlings of both populations, and the lowest lignin content was found in the roots of plants grown in the presence of 2.0 mM Al. Similarly, growth of tea plants in the presence of Al supply reduced lignin concentrations in roots and leaves [9,24] and this delignification was suggested as a stimulus for root elongation and root system development. Inhibition of lignification is related to the enhanced supply of soluble sugars to the roots, which provides energy and stimulates the development and growth of new roots [9]. Development of a larger and less lignified root system may also explain the enhanced uptake of nutrients such as Ca, Mg and P in the presence of Al [9,21].

Al stimulates the production of antioxidant defences in tea plants, which leads to enhanced cell membrane integrity and delayed lignification [21,24] and may contribute to the stimulatory effects of Al on plant growth. The increase in hemicellulose concentration in the leaves of Al treated *M*. *malabathricum* seedlings may explain the increase in leaf thickness of seedlings in this treatment. It is possible that increased hemicellulose concentration and leaf thickness in these plants enhance their physical defences against pathogens and herbivores, which is a current hypothesis for the evolution of plant Al hyperaccumulation [42,43].

Growth with Al in the nutrient solution increased non-structural carbohydrate concentrations, particularly soluble sugars in roots. These findings correspond to previous research on the effects of Al on the amount and distribution of non-structural carbohydrate (NSC) concentrations in tea [9,21]. As in *M*. *malabathricum*, tea plants supplied with Al increase tissue NSC concentrations, and this has been interpreted as a cause of the general growth improvement in response to Al [9]. When tested across a gradient of increasing Al concentration, soluble sugar and starch concentrations in tea leaves initially increase and then decrease after they surpass a distinct threshold Al concentration [21]. Al exposure at 0.3 mM elevates soluble sugar concentrations in tea roots, which is interpreted as a consequence of translocation of carbon from leaves to roots in response to elevated rates of photosynthesis [9]. The increased concentrations of soluble sugars in roots provides energy and inhibits lignification, resulting in new root production and a stimulation of root growth rate [9,24] and nutrient uptake [13,30,44]. Soluble sugars may also include the substrates for production of mucilage and organic acids that contribute to Al uptake and detoxification in Al accumulators [17,19].

These effects of Al addition on NSC concentration in Al accumulators contrast with responses observed in non Al accumulators such as tobacco (*Nicotiana tabacum*), longan (*Dimocarpus longan*) and *Arabidopsis thaliana* [45,46]. For example, although total soluble sugar, reducing sugars and sucrose concentrations all increased in roots, stems and leaves of longan (*Dimocarpus longan*) in response to increasing Al concentrations up to 0.37 mM, they then decreased at higher Al concentrations [47]. In tobacco (*Nicotiana tabacum*), total soluble sugar and starch concentrations in cells declined in response to Al addition [45]. These contrasting results between Al accumulators and non-Al accumulators suggest that stimulation of NSC concentrations in plant tissues in response to exposure to Al is a component of the physiological mechanism that enables expression of high Al concentrations in Al accumulators.

### Trait coordination and stimulation of plant growth by Al

Exposure to low concentrations of Al altered fundamental patterns of physiological trait expression in seedlings of *M*. *malabathricum*. When grown without Al in the nutrient solution, plants were differentiated primarily by the concentrations of both structural and non-structural carbohydrate fractions in roots, and those with higher allocation displayed greater whole-plant K concentrations and faster growth. However inclusion of Al in the nutrient solution generated coordinated changes to the expression of multiple traits that contributed collectively to marked increases in plant growth. Positive correlations among root concentrations of lignin and fibres were retained, but became coupled to differentiation in root starch concentrations, and whole-plant concentrations of P, Ca and Mg. This primary axis of trait variation was orthogonal to a secondary dimension reflecting a coupling of photosynthetic and respiration rates to increasing root concentrations of soluble sugars and whole-plant K concentration. Biomass growth rate was positively associated with higher values along both these dimensions of trait variation, revealing that the growth response to Al was determined by multiple pathways linking up-regulation of gas exchange rates to increased production and allocation of non-structural carbohydrates to roots, changes in other root carbon fractions and enhanced nutrient uptake. These analyses do not reveal the directionality of cause and effect among the pathways linking trait expression to plant growth, but they are consistent with a mechanism that interprets increased sink strength for photosynthates within roots, in response to a demand for carbon metabolites to detoxify Al, as a regulator of photosynthetic activity in source leaves [48].

### Physiological differences between *M*. *malabathricum* populations

Differences in rates of carbon assimilation and partitioning explained the contrasting patterns of growth rate between *M*. *malabathricum* populations. The fast-growing population displayed higher photosynthetic and respiration rates, nutrient concentrations, and concentrations of soluble sugars and starch, fibres and lignin concentrations, than the slow growing population, and in some cases these traits also increased more substantially in response to Al addition in the faster growing population. The ecological drivers and genetic mechanisms that give rise to these population-level differences in physiological and chemical traits remain to be determined, but may relate to local environmental or soil conditions [4,18].

## Conclusions

Exposure to low concentrations of Al benefits the Al accumulator *M*. *malabathrcium* by setting in motion fundamental physiological changes that lead to stimulation of photosynthesis, respiration and growth rates. These changes are associated with enhanced root system development, increases in non-structural carbohydrate concentrations and decreased root system lignification, as well as enhanced uptake of P, Ca and Mg. These responses suggest that enhanced carbon assimilation was triggered to provide the internal resources of non-structural carbohydrates for uptake, transport and storage of Al in this species. However, the scale of physiological and chemical responses to Al application varied between the two populations of *M*. *malabathricum*, and may help to explain their differential response in terms of growth and the maintenance of intraspecific variation in expression of Al accumulation in this species. The close correspondence between these responses presented here for *M*. *malabathricum* and by others for tea [9,21], despite the early divergence of clades containing these species during angiosperm evolution [49], suggests that the underlying physiological mechanisms may have an ancient origin. Research on these Al accumulators highlights the difficulty of interpreting cause and effect from studies showing positive responses of growth and photosynthesis following elemental addition to the growing medium. The classical view is that a positive response in terms of photosynthesis or growth is indicative that the element is ‘limiting’ to plant metabolism [38]. However, Al has no known metabolic function in plants and indeed it is toxic to many plants at high concentrations [8,50,51]. Research on plant Al accumulators reveals that up-regulation of photosynthesis and directed partitioning of the products of photosynthesis to roots is mechanistically linked to detoxification and compartmentalisation of the Al, and this response cannot be interpreted as reflecting a relief from any direct physiological limitation on carbon assimilation. Although this phenomonen has only been demonstrated for plants responding to Al, natural soils often contain multiple elements that may require additional carbon-based metabolites for internal sequestration [38]. This interpretation brings a novel perspective to our understanding of the regulation of carbon assimilation and partitioning in plants and the linkage between these processes and elemental stoichiometry.

## Supporting information

S1 Appendix List of S1-S12 Tables and S1 –S6 Figs in this study. (PDF)
