# Supplementary material for: External aluminium supply regulates photosynthesis and carbon partitioning in the Al-accumulating tropical shrub Melastoma malabathricum
Source: PLoS One. 2024 Mar 20;19(3):e0297686. doi: 10.1371/journal.pone.0297686 (PMC10954143; doi:10.1371/journal.pone.0297686)
Supplement: S1 Appendix — (PDF) [file pone.0297686.s001.pdf]

Supporting Information

Table S1: Mean (±se) concentrations of Al, P, K, Ca and Mg (mg g^{-1}) in leaves, roots and stems of *M. malabathricum* seedlings of a slow-growing population and a fast-growing population grown for 10 weeks in nutrient solutions containing 0 mM, 0.5 mM, 2.0 mM $AlCl_3$.

Part	Pop	Al Treatment	Al (mg g^{-1})	P (mg g^{-1})	K (mg g^{-1})	Ca (mg g^{-1})	Mg (mg g^{-1})
Leaf	slow	0.0 mM	0.31±0.01	6.09±0.4	70.96±3.7	15.07±1.4	6.52±0.7
	slow	0.5 mM	27.25±2.5	6.77±0.3	61.19±2.5	18.04±1.7	6.91±0.6
	slow	2.0 mM	39.40±2.0	6.89±0.3	52.41±2.9	16.93±1.5	6.71±0.3
	fast	0.0 mM	0.41±0.2	7.11±0.6	74.52±2.1	17.91±0.3	6.56±0.5
	fast	0.5 mM	26.40±1.7	8.49±0.7	59.38±2.1	23.09±0.9	9.28±0.7
	fast	2.0 mM	32.94±2.0	9.33±0.7	62.73±2.4	20.96±1.1	8.26±0.7
Root	slow	0.0 mM	0.40±0.1	19.77±2.1	73.42±2.8	5.73±0.7	3.37±0.5
	slow	0.5 mM	46.34±6.7	22.29±1.4	92.74±3.1	4.65±0.7	4.19±0.4
	slow	2.0 mM	84.31±15.6	31.84±1.4	89.76±4.0	4.65±1.0	4.02±0.7
	fast	0.0 mM	0.46±0.1	17.37±1.7	69.61±2.5	5.01±0.5	2.73±0.3
	fast	0.5 mM	50.05±4.9	27.56±2.7	78.92±3.5	4.54±0.2	5.53±0.4
	fast	2.0 mM	109.47±11.0	37.13±3.1	85.92±6.1	4.74±0.2	4.42±0.9
Stem	slow	0.0 mM	0.38±0.2	4.75±0.3	42.78±3.5	10.68±2.4	3.29±0.2
	slow	0.5 mM	12.41±1.2	5.83±0.3	58.67±8.7	16.07±1.3	7.37±1.0
	slow	2.0 mM	14.61±1.3	5.33±0.3	92.20±2.9	15.7±1.5	6.22±1.0
	fast	0.0 mM	0.39±0.1	5.38±0.3	52.37±6.8	9.86±1.4	1.86±0.3
	fast	0.5 mM	8.38±0.4	6.37±0.3	92.21±2.9	14.10±1.5	4.20±1.1
	fast	2.0 mM	12.22±1.7	6.47±0.4	94.81±3.8	16.31±1.8	5.84±1.5

Table S2: Mean square (MS), F Statistics and P values following two way analysis of variance (ANOVA) of P, K, Ca and Mg concentrations in the stems of fast and slow growing populations of *M. malabathricum* seedlings grown for 10 weeks in nutrient solutions containing 0 mM, 0.5 mM, 2.0 mM $AlCl_3$. These values' significance is indicated as follows: *, P < 0.05; **, P < 0.01; ***, P < 0.001.

	P				K			
Factors	df	MS	F Value	P Value	df	MS	F Value	P Value
Treatment	2	3.603	6.005	0.006**	2	5302	25.175	<0.001 ***
Population	1	5.300	8.834	0.005**	1	3058	14.520	<0.001 ***
Treatment: Population	2	0.318	0.529	0.594	2	521	2.470	0.101
Residuals	30	0.600			30	211		

	Ca				Mg			
Factors	df	MS	F Value	P Value	df	MS	F Value	P Value
Treatment	2	113.7	41.147	<0.001 ***	2	26.68	9.210	<0.001 ***
Population	1	4.84	0.431	0.218	1	15.96	5.509	0.025*
Treatment: Population	2	4.85	0.432	0.754	2	7.671	2.648	0.083
Residuals	30	336.8			30	2.897		

Table S3: Mean square (MS), F Statistics and P values following two way analysis of variance (ANOVA) of P, K, Ca and Mg concentrations in the roots of seedlings of fast and slow growing populations of *M. malabathricum* grown for 10 weeks in nutrient solutions containing 0 mM, 0.5 mM, 2.0 mM $AlCl_3$. The significance of these values is indicated as follow: *, P < 0.05; **, P < 0.01; ***, P < 0.001.

	P				K			
Factors	df	MS	F Value	P Value	df	MS	F Value	P Value
Treatment	2	770.0	19.829	<0.001 ***	2	952.3	10.502	<0.001 ***
Population	1	66.6	1.715	0.200	1	458.8	5.060	0.032*
Treatment: Population	2	58.9	1.518	0.236	2	100.3	1.106	0.344
Residuals	30	38.8			30	90.7		

	Ca				Mg			
Factors	df	MS	F Value	P Value	df	MS	F Value	P Value
Treatment	2	2.114	0.859	0.434	2	7.916	5.758	0.008**
Population	1	0.5402	0.291	0.643	1	3.121	2.225	0.145
Treatment: Population	2	0.5301	0.215	0.808	2	1.648	1.174	0.332
Residuals	30	2.461			30	1.399		

Table S4: Mean square (MS), F Statistics and P values following two way analysis of variance (ANOVA) on hemicellulose concentrations (%) in leaves, roots and stems of fast and slow growing populations of *M. malabathricum* grown for 10 weeks in nutrient solutions containing 0 mM, 0.5 mM, 2.0 mM $AlCl_3$. The significance of these values is indicated as follow: *, $P < 0.05$; **, $P < 0.01$; ***, $P < 0.001$.

Part	Leaves				Roots			
Factors	df	MS	F Value	P Value	df	MS	F Value	P Value
Treatment	2	39.09	3.592	0.0399 *	2	46.96	3.090	0.060
Population	1	1.12	0.103	0.7510	1	160.67	10.570	0.002**
Treatment: Population	2	5.19	0.477	0.6254	2	6.79	0.447	0.644
Residuals	30	10.88			30	15.20		

Part	Stem			
Factors	df	MS	F Value	P Value
Treatment	2	15.706	1.076	0.354
Population	1	21.377	1.465	0.236
Treatment: Population	2	5.851	0.401	0.673
Residuals	30	14.596		

Notes: p<0.05*; p<0.005**; p<0.001***

Table S5: Mean square (MS), F Statistics and P values following two way analysis of variance (ANOVA) on cellulose concentrations (%) in leaves, roots and stems of fast and slow growing populations of *M. malabathricum* grown for 10 weeks in nutrient solutions containing 0 mM, 0.5 mM, 2.0 mM $AlCl_3$. The significance of these values is indicated as follow: *, P < 0.05; **, P < 0.01; ***, P < 0.001.

	Leaves				Roots			
Factors	df	MS	F Value	P Value	df	MS	F Value	P Value
Treatment	2	0.595	0.090	0.914	2	44.39	4.609	0.0179*
Population	1	2.884	0.435	0.515	1	144.40	14.994	<0.001 ***
Treatment: Population	2	4.235	0.639	0.535	2	2.07	0.215	0.808
Residuals	30	6.629			30	9.63		

	Stems			
Factors	df	MS	F Value	P Value
Treatment	2	17.544	1.042	0.365
Population	1	9.374	0.557	0.461
Treatment: Population	2	14.960	0.888	0.422
Residuals	30	16.837		

Table S6: Mean square (MS), F Statistics and P values following two way analysis of variance (ANOVA) on lignin concentrations (%) in leaves, roots and stems of fast and slow growing populations of *M. malabathricum* grown for 10 weeks in nutrient solutions containing 0 mM, 0.5 mM, 2.0 mM $AlCl_3$. The significance of these values is indicated as follow: *, P < 0.05; **, P < 0.01; ***, P < 0.001.

	Leaves				Roots			
Factors	df	MS	F Value	P Value	df	MS	F Value	P Value
Treatment	2	0.129	0.058	0.943	2	32.83	14.432	<0.001 ***
Population	1	6.121	2.773	0.106	1	38.27	16.824	<0.001 ***
Treatment: Population	2	1.592	0.721	0.494	2	2.20	0.968	0.391
Residuals	30	2.207			30	2.28		

	Stems			
Factors	df	MS	F Value	P Value
Treatment	2	1.2651	0.520	0.600
Population	1	0.5605	0.230	0.635
Treatment: Population	2	0.635	0.824	0.448
Residuals	30	2.4318		

Table S7: Mean square (MS), F Statistics and P values following two way analysis of variance (ANOVA) on soluble sugar concentrations in leaves, stem and root of *M. malabathricum* seedlings of fast and slow growing populations grown for 10 weeks in nutrient solutions containing 0 mM, 0.5 mM, 2.0 mM $AlCl_3$. The significance of these values is indicated as follow: *, P < 0.05; **, P < 0.01; ***, P < 0.001.

	Leaf				Stem			
Factors	df	MS	F Value	P Value	df	MS	F Value	P Value
Treatment	2	323.2	0.429	0.655	2	728.7	0.650	0.529
Population	1	337.3	0.448	0.509	1	2540.2	2.265	0.143
Treatment: Population	2	1978.1	2.625	0.089	2	338.9	0.302	0.741
Residuals	30	753.6			30	1121.6		

	Root			
Factors	df	MS	F Value	P Value
Treatment	2	8671	7.392	0.002**
Population	1	7957	6.783	0.014*
Treatment: Population	2	1519	1.295	0.288
Residuals	30	1173		

Table S8: Mean square (MS), F Statistics and P values following two way analysis of variance (ANOVA) on starch concentrations in leaf, stem and root of *M. malabathricum* seedlings of fast and slow growing populations grown for 10 weeks in nutrient solutions containing 0 mM, 0.5 mM, 2.0 mM $AlCl_3$. The significance of these values is indicated as follow: *, P < 0.05; **, P < 0.01; ***, P < 0.001.

	Leaf				Stem			
Factors	df	MS	F Value	P Value	df	MS	F Value	P Value
Treatment	2	3.026	0.455	0.639	2	334.7	5.256	0.011*
Population	1	9.404	1.414	0.243	1	14.7	0.231	0.634
Treatment: Population	2	21.325	3.205	0.055	2	24.1	0.378	0.689
Residuals	30	6.653			30	63.7		

	Root			
Factors	df	MS	F Value	P Value
Treatment	2	40.3	0.492	0.616
Population	1	368	4.496	0.042*
Treatment: Population	2	9.6	0.118	0.889
Residuals	30	81.9		

Table S9. Summary of principal components analysis of whole plant nutrient concentrations and physiological variables for two populations of *M. malabathricum* grown without Al in the nutrient solution (0 mM). The significance of these values is indicated as follow: *, P < 0.05; **, P < 0.01; ***, P < 0.001.

	PC1	PC2	PC3	PC4	PC5
Eigenvalue	4.335	2.103	1.734	1.150	0.984
Proportion Explained	0.361	0.175	0.144	0.09585	0.082
Cumulative Proportion	0.361	0.536	0.681	0.777	0.859
Loadings of variables on PC axes					
Elements	PC1	PC2	PC3	PC4	PC5
P	-0.439**	-0.077	0.426**	0.109	-0.672***
K	-0.636***	-0.664***	0.028	0.107	0.263
Ca	-0.305**	0.688***	-0.243	-0.181	-0.399**
Mg	-0.265*	0.248	-0.741***	-0.263	0.222
Photosynthesis	0.213	-0.072	0.654***	-0.568***	0.160
Respiration	-0.247	-0.445	-0.529***	-0.446**	-0.294
Root : Shoot	0.497***	-0.501***	-0.276	0.447**	-0.113
Root soluble sugar	-0.876***	-0.292	-0.044	-0.139	0.061
Root starch	-0.713***	-0.495	0.063	-0.154	-0.136
Root hemicellulose	-0.843***	0.204	-0.006	0.307	0.075
Root cellulose	-0.822***	0.338	0.030	0.323	0.100
Root lignin	-0.612***	0.301	0.284	-0.117	0.226

Table S10. Output from a general linear model fitting data on final dry mass for plants from two populations of *M. malabathricum* grown without Al addition to the nutrient solution to their scores along the first two axes of a PCA describing variation in 12 traits.

	Estimate	Std Error	t Value	p value
Intercept	2.9567	0.1839	16.078	6.16e-08 ***
PC axis 1	-0.5492	0.1877	-2.926	0.0169 *
PC axis 2	-0.2313	0.1879	-1.231	0.2495

Table S11. Summary of principal components analysis of whole plant nutrient concentrations and physiological variables for two populations of *M. malabathricum* grown with Al in the nutrient solution (0.5 or 2.0 mM $AlCl_3$). The significance of these values is indicated as follow: *, P < 0.05; **, P < 0.01; ***, P < 0.001.

	PC1	PC2	PC3	PC4	PC5
Eigenvalue	3.947	2.782	1.336	0.9302	0.848
Proportion Explained	0.328	0.232	0.113	0.0775	0.071
Cumulative Proportion	0.329	0.560	0.672	0.7496	0.820
<u>Loadings of variables on PC axes</u>					
P	0.713***	0.353**	-0.450***	0.102	-0.538***
K	0.373**	0.608***	-0.272	-0.611***	0.588***
Ca	0.899***	-0.261	0.073	0.294	0.301*
Mg	0.847***	0.218	-0.514***	-0.222	-0.361**
Photosynthesis	0.410***	0.689***	0.432***	0.549***	0.038
Respiration	-0.196	-0.860***	0.363**	-0.159	-0.344*
Root : Shoot	0.328**	0.195	0.788***	-0.399***	-0.154
Root soluble sugar	0.381**	0.909***	0.140	0.255**	-0.101
Root starch	0.659***	0.401**	0.528***	-0.367***	-0.098
Root hemicellulose	0.906***	-0.591***	0.123	-0.069	-0.137
Root cellulose	0.909***	-0.582***	-0.193	-0.081	0.088
Root lignin	0.870***	-0.561***	0.112	0.267	0.359**

Table S12. Output from a general linear model fitting data on final dry mass for plants from two populations of *M. malabathricum* grown with Al in the nutrient solution (0.5 or 2.0 mM $AlCl_3$) to their scores along the first two axes of a PCA describing variation in 12 traits.

	Estimate	Std Error	t Value	p value
Intercept	4.2848	0.1752	24.455	<2e-16 ***
PC axis 1	0.5064	0.2108	2.402	0.0256 *
PC axis 2	0.4836	0.2107	2.295	0.0322 *

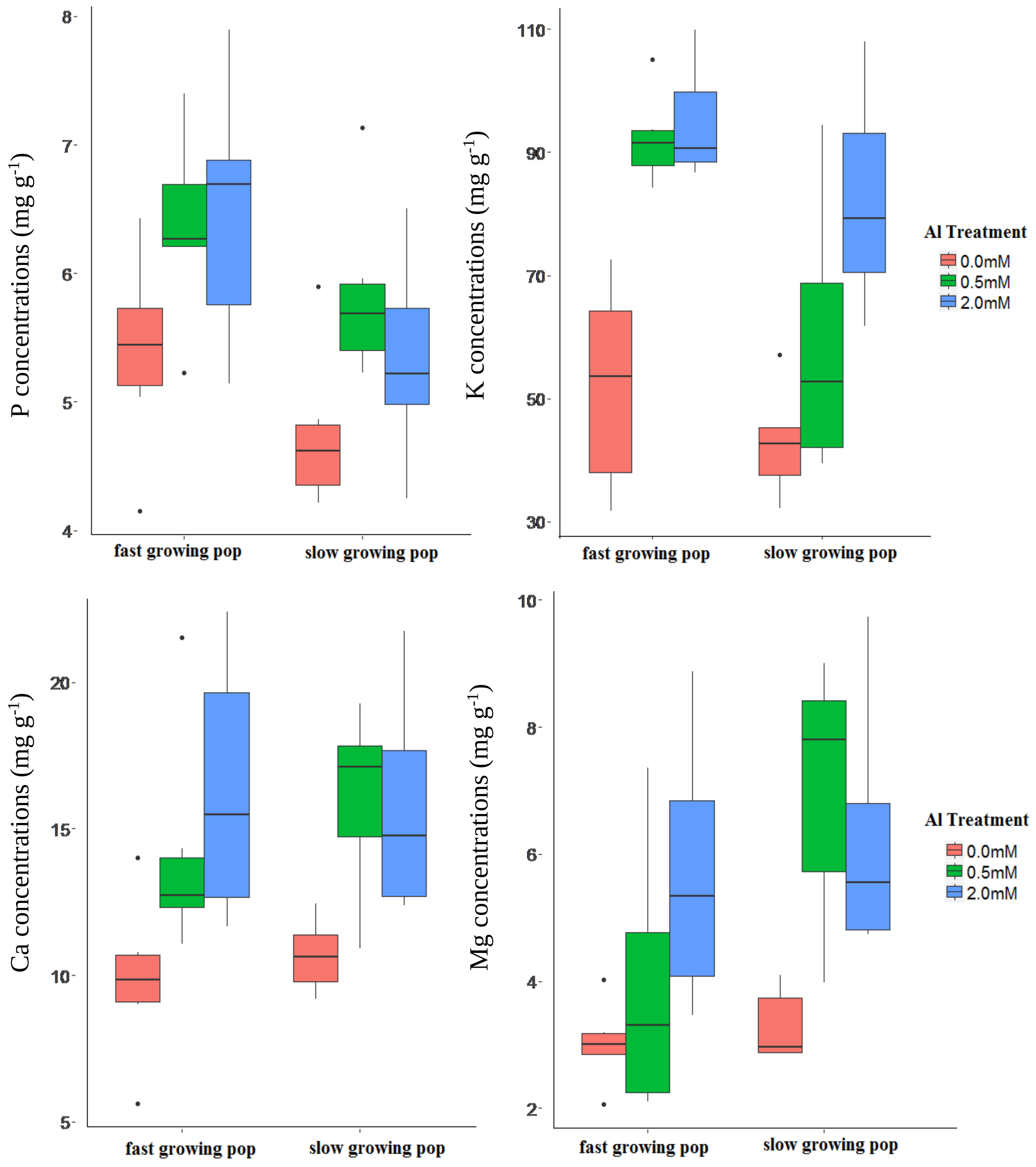

Fig. S1: Boxplots of concentrations of P, K, Ca and Mg (mg g^{-1}) in the stems of fast and slow growing populations of *M. malabathricum* seedlings grown for 10 weeks in nutrient solutions containing 0 mM, 0.5 mM, or 2.0 mM $AlCl_3$.

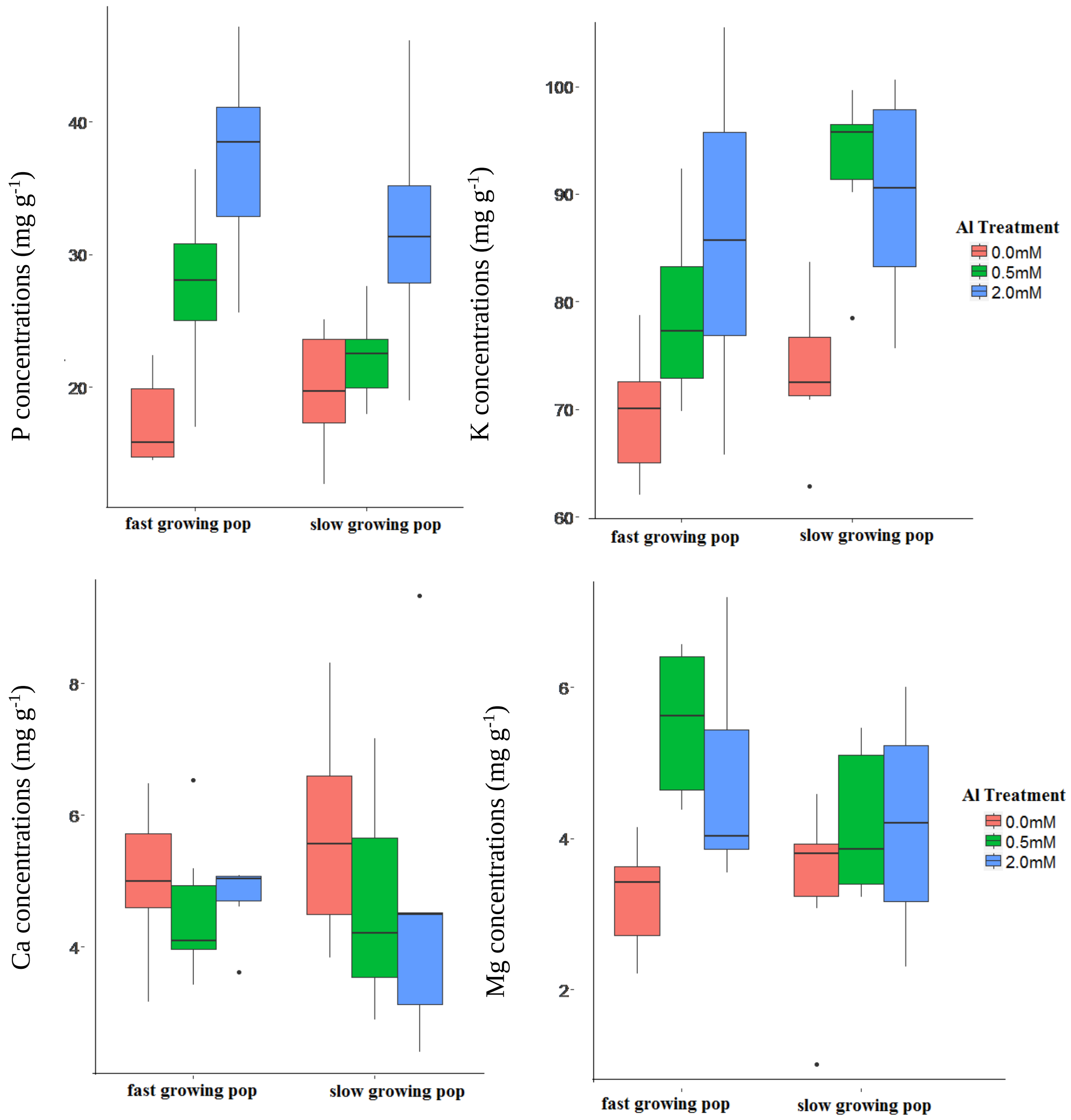

Fig. S2: Boxplots of concentrations of P, K, Ca and Mg (mg g^{-1}) in the roots of fast and slow growing populations of *M. malabathricum* seedlings grown for 10 weeks in nutrient solutions containing 0 mM, 0.5 mM, or 2.0 mM $AlCl_3$.

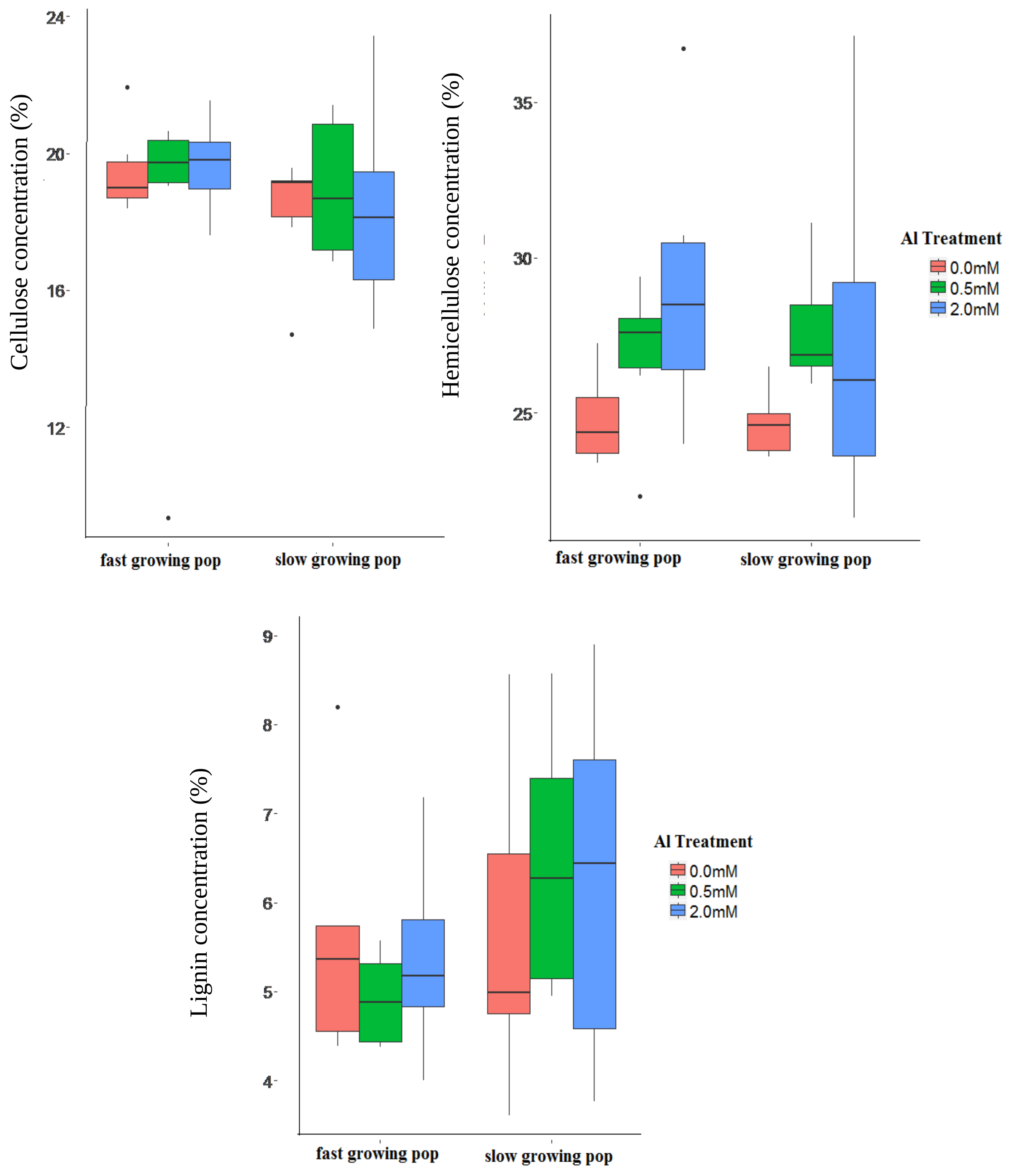

Fig. S3: Boxplots of concentrations of cellulose, hemicellulose and lignin (%) in the leaves of fast and slow growing populations of *M. malabathricum* seedlings grown for 10 weeks in nutrient solutions containing 0 mM, 0.5 mM, or 2.0 mM $AlCl_3$

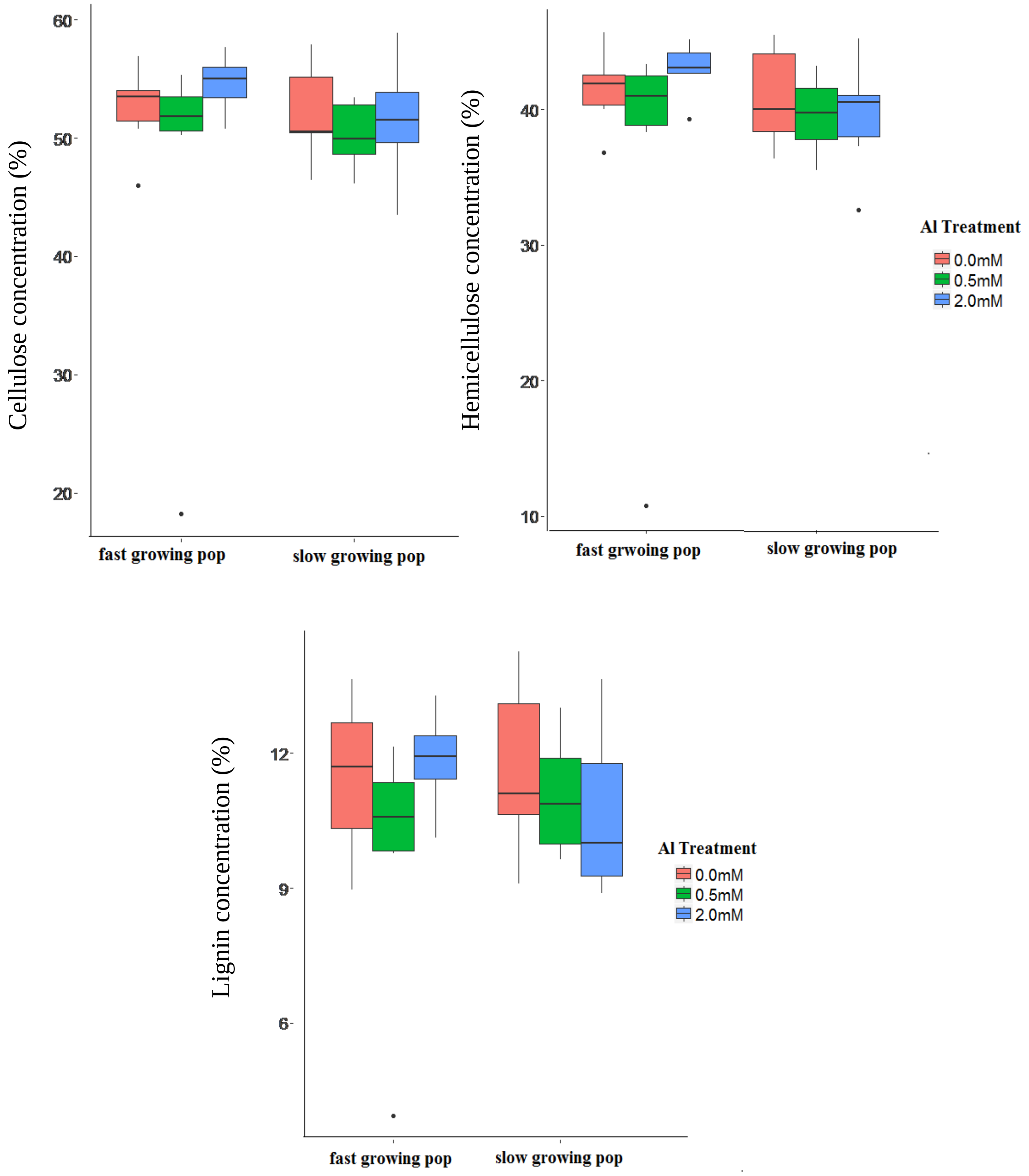

Fig. S4: Boxplots of concentrations of cellulose, hemicellulose and lignin (%) in stems of fast and slow growing populations of *M. malabathricum* seedlings grown for 10 weeks in nutrient solutions containing 0 mM, 0.5 mM, or 2.0 mM $AlCl_3$

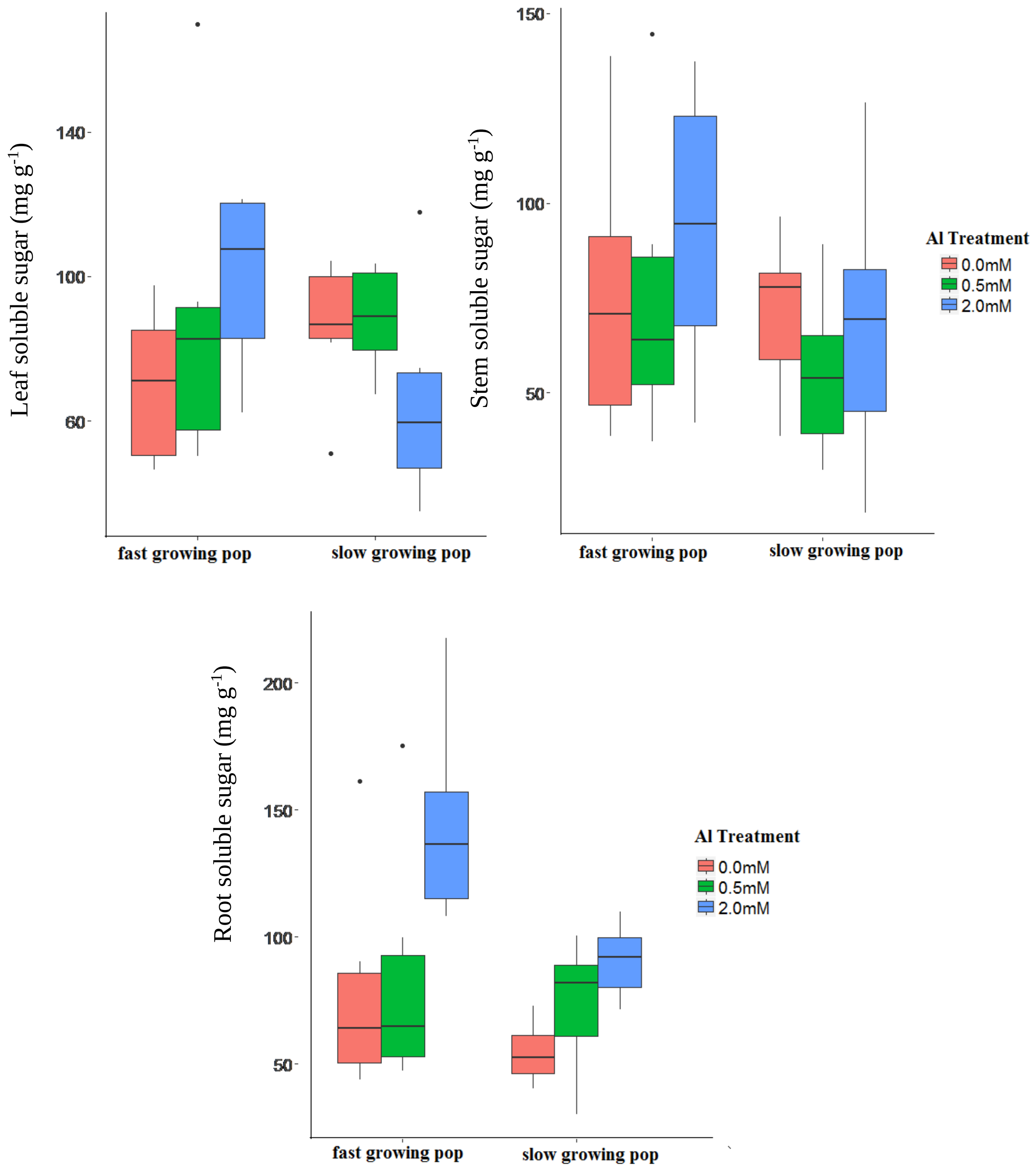

Fig. S5: Boxplots of concentrations of soluble sugars (mg g^{-1}) in leaves, stems and roots of seedlings of slow and fast growing populations of *M. malabathricum* grown for 10 weeks in nutrient solutions containing 0 mM, 0.5 mM, 2.0 mM $AlCl_3$.

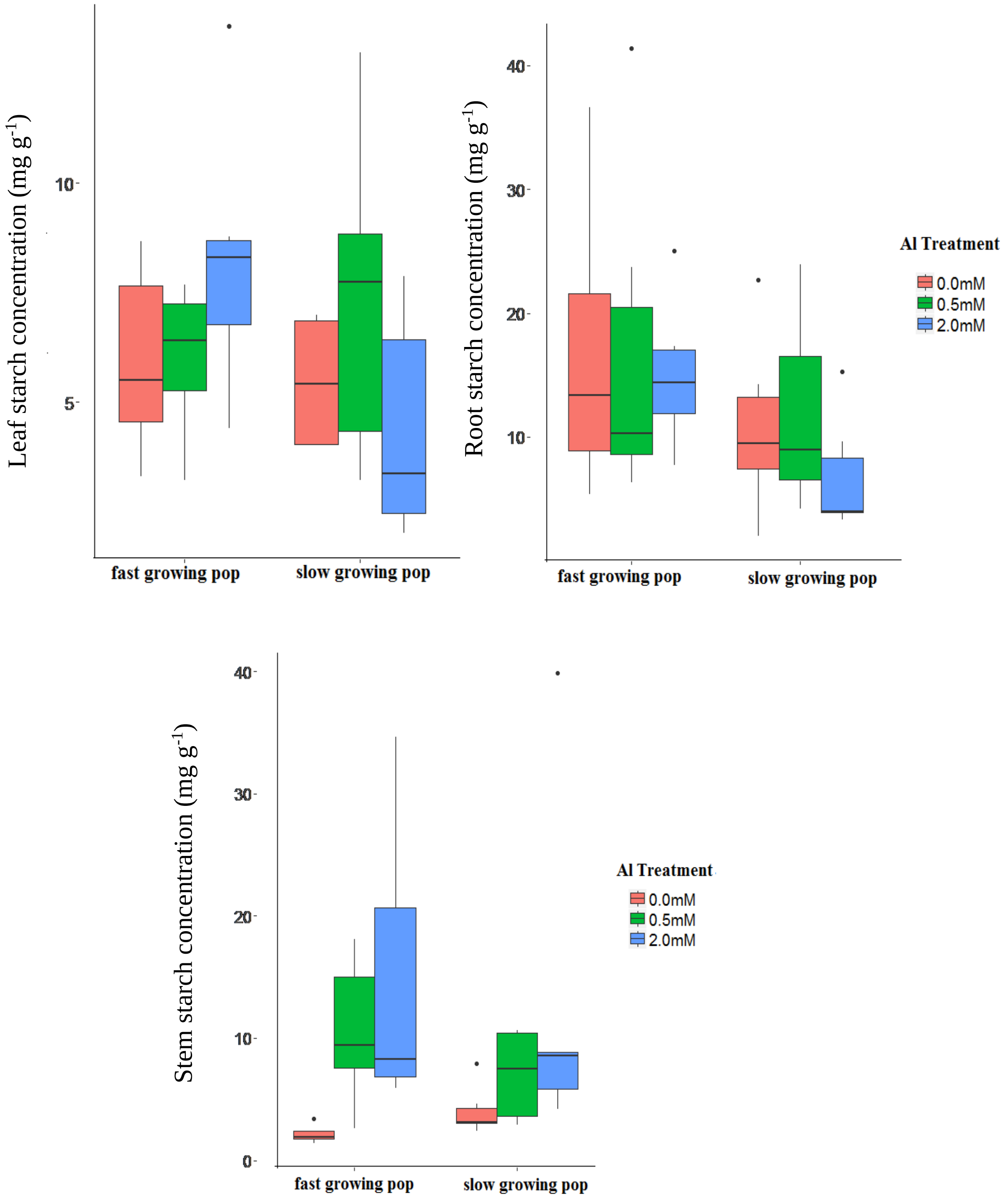

Fig. S6: Boxplots of concentrations of starch in leaves, stems and roots (mg g^{-1}) of slow and fast growing populations of *M. malabathricum* grown for 10 weeks in nutrient solutions containing 0 mM, 0.5 mM, 2.0 mM $AlCl_3$.
